# Chidamide Is Screened to Suppress Epileptogenesis in Mice Models via Blocking Histone Deacetylase 1

**DOI:** 10.1002/cns.70805

**Published:** 2026-02-23

**Authors:** Qian Guo, Zhao‐Jun Wang, Wei‐Bo Dong, Li Pang, Xiao‐Yuan Mao

**Affiliations:** ^1^ Department of Clinical Pharmacology, Hunan Key Laboratory of Pharmacogenetics and National Clinical Research Center for Geriatric Disease (Xiangya Hospital) Xiangya Hospital Central South University Changsha China; ^2^ Institute of Clinical Pharmacology and Engineering Research Center of Applied Technology of Pharmacogenomics of Ministry of Education Central South University Changsha China

**Keywords:** chidamide, epilepsy, epileptogenesis, histone deacetylase 1

## Abstract

**Aims:**

Disease‐modifying and anti‐epileptogenic therapies for epilepsy remain limited. Given the critical role of histone deacetylases (HDACs) in epileptogenesis, this study aimed to identify effective HDAC inhibitors and evaluate their anti‐epileptogenic potential.

**Methods:**

A Mg^2+^‐free neuron‐like PC12 cell model was used to screen FDA‐approved HDAC inhibitors in vitro. Acute and chronic epilepsy models were induced in mice using pentylenetetrazol (PTZ) or kainic acid (KA). Spontaneous recurrent seizures (SRS) were monitored by electroencephalogram (EEG). Neuronal survival, mossy fiber sprouting (MFS), and glial activation were assessed by Nissl staining, Timm staining, and immunofluorescence, respectively. HDAC1 expression was analyzed by Western blot and immunofluorescence. Neuron‐specific HDAC1 overexpression was achieved using adeno‐associated virus (AAV2; hereafter referred to as AAV)‐mediated gene transfer.

**Results:**

Chidamide (Chi) exhibited the most inhibitory effect among nine HDAC inhibitors. Chi significantly reduced seizure susceptibility in acute PTZ‐ and KA‐induced models. In the chronic KA model, Chi attenuated SRS, improved neuronal survival, reduced MFS, and suppressed astrocytic and microglial activation. Chi markedly decreased hippocampal HDAC1 expression, while neuronal HDAC1 overexpression abolished its anti‐epileptogenic effects.

**Conclusion:**

Chi attenuates epileptogenesis by inhibiting neuronal HDAC1 and may serve as a promising repurposed anti‐epileptogenic therapy.

## Graphical abstract

Proposed model of the anti‐epileptogenic actions of chidamide. Chidamide mitigates drug‐induced seizure severity during the early stage of epileptogenesis, suppresses status‐epilepticus‐driven spontaneous recurrent seizures, and alleviates key pathological hallmarks, including mossy fiber sprouting, neuronal loss, and reactive astrocytic and microglial proliferation in the middle stage.

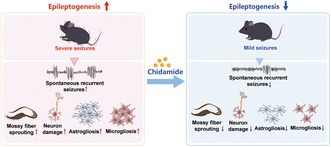

**Abbreviations**
AP: Anteroposterior
ASM: Anti‐seizure medication
Chi: Chidamide
DG: Dentate gyrus
DMSO: Dimethylsulfoxide
DV: Dorsoventral
EEG: Electroencephalogram
FDA: Food and Drug Administration
GFAP: Glial fibrillary acidic protein
HDAC: Histone deacetylase
HRP: Horseradish peroxidase
KA: Kainic acid
MFS: Mossy fiber sprouting
ML: Mediolateral
PBS: Phosphate‐buffered saline
PFA: Paraformaldehyde
PTZ: Pentylenetetrazol
SAHA: Vorinostat
SRS: Spontaneous recurrent seizures
TBST: Tris buffered saline tween
VPA: valproic acid

## 1 Introduction

Epilepsy is a chronic brain disorder characterized by the appearance of long‐standing unprovoked seizures [1]. Globally, there is approximately 1% of the population across all age groups [2] with the estimated number of 70 million patients who suffer from epilepsy. Apart from epileptic seizures, diverse comorbidities like cognitive deficits, anxiety, and depression also occur, which have serious effects on life quality [3]. To date, more than 35 anti‐seizure medications (ASMs) have been approved for seizure control in clinical practice [4, 5]. By manipulating synaptic excitability, these agents have the ability to achieve symptomatic relief [6], sometimes requiring long‐term or even lifelong administration. However, their efficacy is limited, with a stable drug resistance rate of ~30% [7]. Furthermore, the currently applicable ASMs merely ameliorate epileptic seizures but do not halt the epileptogenic process [8], which results in relapse and becomes drug‐resistant epilepsy. Therefore, it is of urgent need to explore an anti‐epileptogenic approach to treat epilepsy.

Over the past two decades, there has been increasing attention toward epileptogenesis—the process involving molecular, cellular, and network alterations that transform a healthy brain into one capable of generating spontaneous seizures [9, 10]. This process, initiated by diverse insults such as traumatic brain injury, status epilepticus, or genetic mutations, encompasses a latent period during which neural circuitry undergoes maladaptive plasticity, inflammation, and gliosis [11, 12]. Targeting epileptogenesis offers a conceptual shift from symptom control to disease modification, with the potential to prevent or delay epilepsy onset and reduce its long‐term burden [13, 14, 15]. In contrast to conventional ASMs, which act on hyperexcitable networks during seizures, anti‐epileptogenic strategies aim to interrupt the pathophysiological cascade that sustains and amplifies epileptic networks [14]. Identifying and validating effective interventions in this preclinical window represents a critical frontier in epilepsy research and holds promise for improving patients' outcomes.

Among emerging therapeutic targets, histone deacetylases (HDACs) have attracted considerable interest. As key epigenetic regulators, HDACs modulate gene expression and chromatin structure by removing acetyl groups from histone and non‐histone proteins, thus influencing neuronal excitability, synaptic plasticity, and neuroinflammatory pathways [16, 17]. Recent evidence emphasizes the crucial role of aberrant HDAC activity in epileptogenesis, and HDAC inhibition has shown strong, potent disease‐modifying effects in preclinical models [18, 19]. For instance, administration of the HDAC inhibitor sodium butyrate in a hippocampal kindling model was previously reported to significantly delay the development of temporal lobe epilepsy and reduce mossy fiber sprouting (MFS), indicating both prevention and reversal of epileptogenic networks [20]. Additionally, a comprehensive review further substantiates HDAC dysregulation in epilepsy and discusses the feasibility of targeting HDAC isoforms for therapeutic intervention [21]. Critically, several HDAC inhibitors, including chidamide (Chi), valproic acid (VPA), vorinostat (SAHA), romidepsin, belinostat, panobinostat, divalproex sodium, givinostat, and sodium phenylbutyrate, are successfully utilized for various oncological indications and have well‐established safety profiles. These evidences give the hint that repurposing these compounds for anti‐epileptogenic therapy is a rational strategy to treat epilepsy.

On the basis of this rationale, our present work revealed that Chi was screened to potently abrogate neuronal activity and improve neuronal survival via phase contrast, using an FDA‐approved HDAC inhibitor library. Furthermore, our present results revealed that treatment with Chi remarkably ameliorated seizure susceptibility in pentylenetetrazol (PTZ)‐ and kainic acid (KA)‐induced epilepsy mouse models. Notably, the anti‐epileptogenic effect of Chi was also found in the KA‐induced epilepsy mouse model, possibly via inhibition of HDAC1.

## 2 Materials and Methods

### 2.1 Animals

Adult male C57BL/6J mice (6–8 weeks old, 18–22 g), provided by the Animal Center of Central South University (Hunan, China), were housed under standardized conditions (24°C ± 2°C, 12‐h light/dark cycle). Food and water were provided ad libitum, and animals were kept free from external disturbances throughout the experimental period.

### 2.2 Drug Treatments

Experiment 1: To evaluate the effect of Chi on seizure susceptibility in an acute PTZ‐induced seizure model, male C57BL/6J mice were randomly assigned to five groups (*n* = 6 per group): (1) PTZ group: Mice were pretreated with subcutaneous (s.c.) injections of vehicle (saline with 2% DMSO) once daily for 6 consecutive days, followed by a single intraperitoneal (i.p.) injection of PTZ (70 mg/kg in saline) on Day 7; (2) 2.5 mg/kg/2d Chi + PTZ group: Mice received Chi (2.5 mg/kg, s.c.) every other day for 6 days (three injections in total), followed by PTZ (70 mg/kg, i.p.) on Day 7; (3) 2.5 mg/kg/d Chi + PTZ group: Mice received Chi (2.5 mg/kg, s.c.) once daily for 6 consecutive days, followed by PTZ (70 mg/kg, i.p.) on Day 7; (4) 5 mg/kg/2d Chi + PTZ group: Mice received Chi (5 mg/kg, s.c.) every other day for 6 days, followed by PTZ on Day 7; (5) 5 mg/kg/d Chi + PTZ group: Mice received Chi (5 mg/kg, s.c.) once daily for 6 consecutive days, followed by PTZ on Day 7. Chi was dissolved in 2% dimethylsulfoxide (DMSO)‐saline before administration. All PTZ injections were performed using a freshly prepared solution at a dose of 70 mg/kg.

Experiment 2: To investigate the inhibitory effect of Chi on epileptogenesis, mice were randomly divided into five experimental groups (*n* = 6 per group): (1) Con group: Mice received a unilateral stereotaxic injection of 1 μL phosphate‐buffered saline (PBS) into the right hippocampus. Fourteen days post‐injection, mice were treated with daily vehicle injections (s.c.) for 14 consecutive days; (2) KA group: Mice received a 1 μL stereotaxic injection of KA (250 ng/μL dissolved in saline) into the right hippocampus. Starting 14 days post‐KA, animals received daily vehicle injections (s.c.) for 14 consecutive days; (3) KA + 5 mg/kg/2d Chi group: Mice were injected with KA as described above. Fourteen days later, Chi (5 mg/kg, s.c.) was administered once every other day over a 14‐day period (total of 7 injections). (4) KA + 5 mg/kg/d Chi group: Following KA injection, mice received daily s.c. injections of Chi (5 mg/kg) for 14 consecutive days, starting 14 days post‐KA; (5) 5 mg/kg/d Chi group: Mice received a unilateral injection of 1 μL PBS, followed 14 days later by daily s.c. injections of Chi (5 mg/kg) for 14 consecutive days.

Experiment 3: To evaluate whether neuronal HDAC1 overexpression could reverse the anti‐epileptogenic efficacy of Chi, mice were randomly assigned to the following three groups (*n* = 6 per group): (1) AAV‐NC + Sub‐KA group: Mice received a unilateral stereotaxic injection of 0.598 μL AAV‐NC into the right hippocampus. After 3 weeks, 1 μL of PBS was injected into the same site. Fourteen days later, mice were administered daily injections (s.c.) of vehicle for 14 consecutive days; (2) AAV‐HDAC1 + Sub‐KA group: Mice received a unilateral injection of 0.598 μL AAV‐HDAC1. After 3 weeks, a sub‐convulsive dose of KA (Sub‐KA; 100 ng/μL in saline, 1 μL) was injected into the hippocampus. Starting 14 days post‐KA, mice received daily vehicle injections (s.c.) for 14 consecutive days; (3) AAV‐HDAC1 + Sub‐KA + Chi group: Mice were injected with 0.598 μL AAV‐HDAC1 into the hippocampus. Three weeks later, they received a 1 μL subconvulsive dose of KA (100 ng/μL). Fourteen days after KA injection, mice were treated daily with Chi (5 mg/kg, s.c.) for 14 consecutive days.

### 2.3 PTZ‐Induced Seizure Mouse Model

PTZ was freshly dissolved in saline with the stock concentration at 70 mg/kg. Mice received a single intraperitoneal (i.p.) injection of PTZ to induce seizures. Following injection, animals were individually placed in transparent observation cages and continuously monitored for 30 min according to the Racine Score as elaborated in Section 2.6.

### 2.4 KA‐Induced Epilepsy Mouse Model

Mice were anesthetized with sodium pentobarbital (50 mg/kg, i.p.) and placed in a stereotaxic apparatus (RWD Life Science). A microsyringe connected to an infusion pump (62,204, RWD Life Science) delivered 1 μL KA (250 ng/μL in saline; K0250, Sigma‐Aldrich) into the right hippocampus (AP −2.0 mm, ML −1.8 mm, DV −2.3 mm) at 0.20 μL/min. The needle was left in place for 5 min to prevent reflux. Controls received 1 μL saline. Seizure severity was assessed within 90 min using Racine Score; mice with a stage more than seizure stage 3 were included as KA‐treated epileptic models. Two weeks post‐KA, mice were administered Chi (5 mg/kg, s.c.) or vehicle (saline with 2% DMSO) once daily or every other day for 14 days, according to group allocation. After treatment, a continuous Electroencephalogram was recorded for 5 days. Animals were then perfused for subsequent histological and Immunofluorescence and Timm staining.

### 2.5 AAV Injection

Overexpression of HDAC1 in hippocampal neurons was achieved via adeno‐associated virus (AAV2; hereafter referred to as AAV)‐mediated gene transfer. AAV vectors (serotype 2, titer 1 × 10^12^ vg/mL; HANBIO, Shanghai, China) carrying mouse HDAC1 ( *Mus musculus* , NCBI RefSeq accession no. NM_008228) under the control of the synapsin promoter (AAV‐Syn‐Hdac1‐ZsGreen‐3Flag) were used. The negative control (NC) virus consisted of the same AAV backbone expressing ZsGreen alone without the Hdac1 coding sequence under the synapsin promoter (AAV‐Syn‐NC‐ZsGreen‐3Flag). After anesthesia, mice were placed in a stereotaxic apparatus (RWD Life Science). A microinfusion pump (R452; RWD Life Science) was used for virus delivery. Each mouse received a unilateral injection into the right hippocampus (AP −2.00 mm, ML −1.80 mm, DV −2.30 mm from bregma) at a rate of 46 nL/min, with a total volume of 0.598 μL. The needle was left in place for an additional 5 min post‐infusion to minimize reflux, and the scalp was sutured after completion. Three weeks after injection, four mice per group were sacrificed under deep anesthesia. Hippocampal tissues were collected from the virus‐injected hemisphere for immunofluorescence and Western blot analysis to verify HDAC1 overexpression in neurons.

### 2.6 Racine Score

Behavioral seizures were assessed in a blinded manner for 30 min following PTZ administration or 90 min after KA injection. Seizure severity was scored according to the Racine scale: stage 0, no response; stage 1, facial and vibrissae twitching; stage 2, head nodding and circling; stage 3, myoclonic jerks involving forelimbs and hindlimbs; stage 4, rearing with loss of posture; stage 5, generalized tonic–clonic seizures accompanied by running and jumping; and stage 6, death. Mice that exhibited more than stage 3 seizure were considered to have experienced convulsive events and were included in subsequent analyses.

### 2.7 Electroencephalogram (EEG) Recording

To evaluate spontaneous recurrent seizures (SRS) during epileptogenesis, mice underwent cortical EEG recordings for 2 h per day over 5 consecutive days. One week prior to EEG monitoring, mice were anesthetized and implanted with cortical electrodes using a stereotaxic apparatus. A recording electrode was positioned over the cortex (AP −2.0 mm, ML −1.8 mm, DV −2.3 mm), and additional screws were placed to serve as ground and reference. Electrodes were secured with dental cement and connected to a micro‐connector. After a 7‐day recovery period, EEG recordings were initiated. EEG data were acquired and analyzed using Sirenia Seizure software (Pinnacle Technology). SRS events were identified as rhythmic spike–wave discharges lasting > 10 s, with a frequency > 3 Hz and amplitude at least twice that of the baseline. Daily SRS frequency was quantified for each mouse.

### 2.8 Nissl Staining

After deparaffinization and rehydration, paraffin‐embedded brain sections (5 μm thickness) were placed in a dark box. Approximately 500 μL of Nissl staining solution (Beyotime, China) was added to fully cover each section. The slides were then incubated in a biochemical incubator at 37°C for 10 min. Following incubation, the sections were gently rinsed back and forth with distilled water using a Pasteur pipette to remove excessive staining solution. The sections were then immersed in 70% ethanol for 2 s to slightly differentiate the staining and air‐dried completely at room temperature. After drying, the slides were cleared in xylene I and xylene II, 5 min each. Excessive xylene around the sections was carefully removed, and a coverslip was applied using neutral resin for mounting. Images were captured using a light microscope. For quantitative analysis, the number of neurons with clearly visible Nissl bodies was counted using ImageJ software (NIH, USA).

### 2.9 Timm Staining

MFS in epilepsy was assessed by Timm staining. Briefly, mice were deeply anesthetized and transcardially perfused with 20 mL of saline, followed by 50 mL of 0.4% sodium sulfide (in 0.1 M phosphate buffer), and 20 mL of 4% paraformaldehyde. Brains were removed, post‐fixed in 4% paraformaldehyde overnight, and cryoprotected in 30% sucrose for 3 d. Coronal frozen sections of the hippocampus were prepared. Timm staining solution was freshly prepared by mixing equal volumes of the following: 50% gum Arabic, citric acid buffer (2.55 g citric acid and 2.35 g trisodium citrate in 10 mL of water), 5.67% hydroquinone, and 0.73% silver lactate. Solutions were protected from light and mixed immediately before use. Sections were air‐dried and incubated with the staining solution under the dark environment for 90–120 min. Afterward, sections were rinsed in running tap water, dehydrated, cleared, and mounted with neutral balsam. Images were acquired using a light microscope.

### 2.10 Immunofluorescence

For immunofluorescent staining of brain slices (5 μm thickness), mice were deeply anesthetized and perfused transcardially with saline followed by 4% PFA. Brains were post‐fixed in 4% PFA overnight at 4°C and then embedded in paraffin. Sections were deparaffinized in xylene and rehydrated through a graded ethanol series. Antigen retrieval was performed by heating sections in citrate buffer (pH 6.0) at 100°C for 20 min. After cooling and rinsing with PBS, sections were permeabilized with 0.5% Triton X‐100 for 15 min and blocked with 10% normal donkey serum for 30 min at room temperature. Sections were incubated with the following primary antibodies overnight at 4°C: mouse anti‐NeuN (1:200; HUABIO), mouse anti‐GFAP (1:100; Beyotime), rabbit anti‐Iba1 (1:100; ABclonal), and rabbit anti‐HDAC1 (1:200; Abcam). After washing, sections were incubated with Alexa Fluor 488‐ or 555‐conjugated secondary antibodies (1:200; Thermo Fisher) for 1 h at room temperature in the dark. Nuclei were counterstained with DAPI, and slides were mounted using antifade mounting medium. Fluorescence intensity and positive cell counting were analyzed using ImageJ software.

### 2.11 Western Blotting

Hippocampal tissues were harvested and lysed in RIPA buffer (P0013B; Beyotime Biotechnology, China), supplemented with phosphatase inhibitor A (P1045; Beyotime Biotechnology, China), phosphatase inhibitor B (P1045; Beyotime Biotechnology, China), and protease inhibitor (P1005; Beyotime Biotechnology, China) at a ratio of 100:1:1:1 (v/v/v/v). Cell and tissue lysates were sonicated for 30 s. The supernatants were retained and subsequently quantified using a BCA protein assay kit (P0006; Beyotime Biotechnology, China). In brief, 20 μg proteins for each sample were subjected to sodium dodecyl sulfate–polyacrylamide gel electrophoresis and then electrophoretically transferred to polyvinylidene fluoride membranes. After blocking with TBST containing 5% nonfat milk for 1 h, the membranes were incubated with anti‐HDAC1 (rabbit, 1:5000; Abcam), anti‐GAPDH (mouse, 1:1000; Beyotime Bio‐technology) overnight at 4°C. The next day, following three washes in TBST, the membranes were incubated with horseradish peroxidase (HRP)‐conjugated IgG (rabbit, A9169, 1:10,000; Sigma) or anti‐mouse IgG (mouse, A9044, 1:10,000; Sigma) at room temperature for 1 h. The protein band intensity was quantified by using ImageJ software. The protein expression levels were normalized to GAPDH.

### 2.12 Statistics

All results in this study are presented as mean ± SEM. Statistical analyses were performed using GraphPad Prism 9.0 software. Prior to group comparisons, the Shapiro–Wilk test was applied to assess the normality of data distribution. For comparisons between two groups, unpaired *t*‐tests were conducted for normally distributed data, while the Mann–Whitney *U* test was used for non‐normally distributed data. For comparisons among multiple groups, one‐way analysis of variance (one‐way ANOVA) followed by Tukey's post hoc test was performed for normally distributed datasets. In cases where data did not meet normality assumptions, the Kruskal–Wallis nonparametric test was used. A *p*‐value < 0.05 was considered statistically significant.

Animal numbers (*n* = 5–6 per group) were based on prior studies using the KA‐induced chronic epilepsy model [22]. While a formal prospective power analysis was not performed, prior experience and observed large effects on seizure‐related outcomes indicated that these group sizes were sufficient to achieve high statistical power.

## 3 Results

### 3.1 Chi Was Screened to Potently Improve Neuronal Survival and Reduce Seizure Susceptibility in a PTZ‐Induced Acute Seizure Model

**FIGURE 1:**
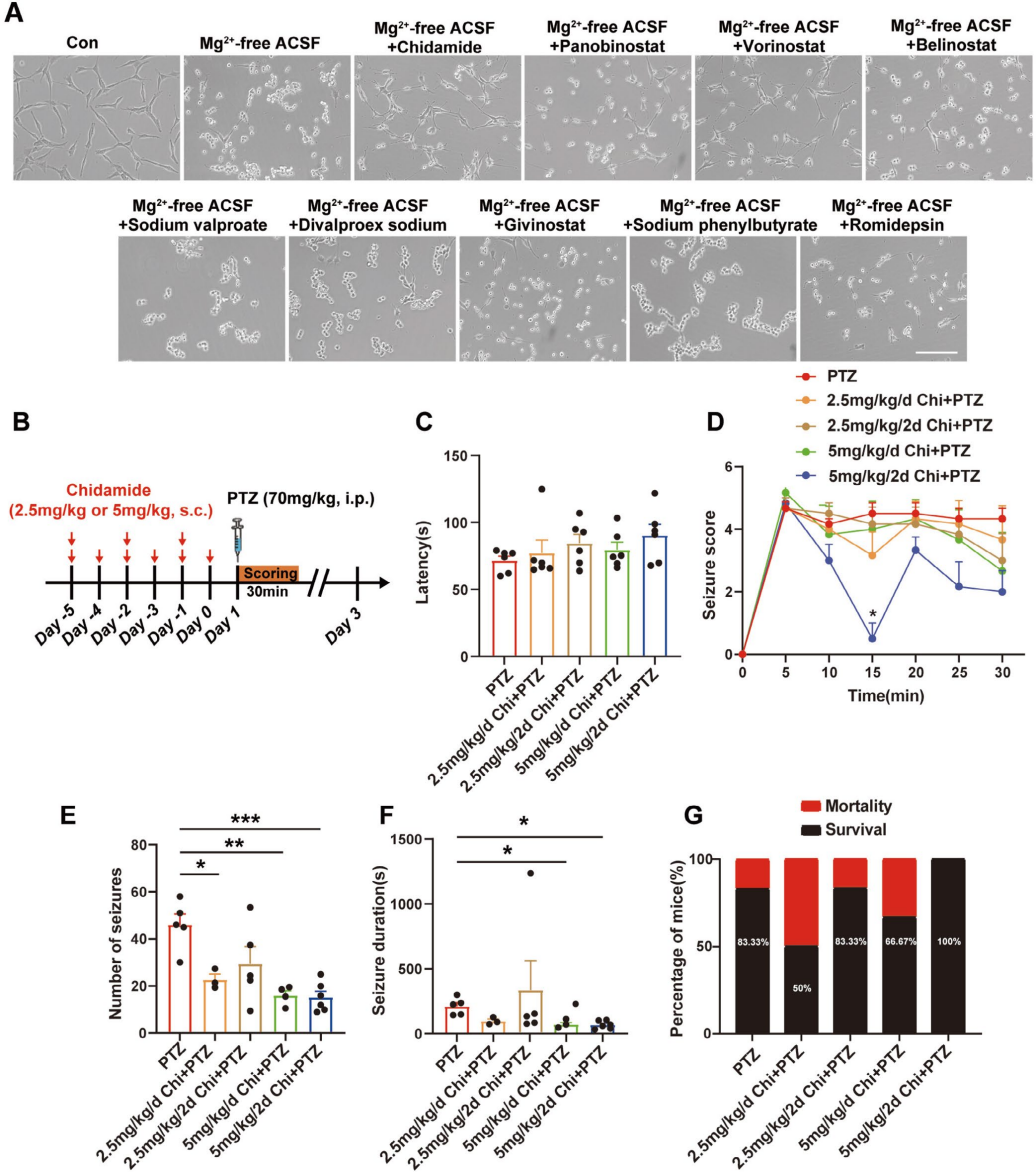
Chi was screened to potently improve neuronal survival and reduce seizure susceptibility in a PTZ‐induced acute seizure model. (A) Representative images for evaluating the effects of nine FDA‐approved HDAC inhibitors on neuronal survival via phase contrast in different groups. scale bar: 50 μm. (B) Schematic of the experimental protocol, including PTZ induction and chidamide treatment. (C) Effect of chidamide on seizure latency. (D) Effect of chidamide on seizure severity. (E) Effect of chidamide on seizure frequency. (F) Effect of chidamide on seizure duration. (G) Effect of chidamide on the survival rate in PTZ‐treated mice. *p < 0.05, **p < 0.01, ***p < 0.001.

To identify HDAC inhibitors with potential neuroprotective effects, we screened a panel of FDA‐approved compounds, including nine HDAC inhibitors, in a Mg^2+^‐free‐induced PC12 cell model. Phase‐contrast microscopy was employed to assess cell survival. Chi treatment substantially improved neuronal survival in comparison with that in the Mg^2+^‐free model (Figure 1A). These findings suggest that Chi improves neuronal survival in a Mg^2+^‐free‐induced PC12 cell model. The timeline evaluating the anti‐seizure potential of Chi is displayed in Figure 1B. It was found that pretreatment with Chi at a dose of 5 mg/kg every other day resulted in a decrease of seizure score over time in the PTZ model group (Figure 1D), despite no significance being observed in the facet of seizure latency (Figure 1C). Notably, a pronounced transient decrease in seizure score was observed at the 15‐min time point in the 5 mg/kg every‐other‐day group compared with daily dosing (*p* < 0.05). This effect was specific to this early time point and was consistently observed across independent experiments. The transient nature of this response may reflect a short‐term peak pharmacodynamic effect associated with intermittent dosing. Both daily and every‐other‐day administration of Chi reduced PTZ‐induced seizure severity. Quantitative analysis further showed that Chi significantly decreased seizure frequency in a dose‐dependent manner (Figure 1E). In addition, seizure duration was also significantly reduced in PTZ‐induced seizure mice treated with Chi, with the alternate‐day treatment producing a more pronounced effect (Figure 1F). Importantly, Chi pretreatment also improved animals' survival following PTZ‐induced seizures (Figure 1G), suggesting a protective effect against seizure‐induced death. Taken together, these results indicate that Chi effectively reduces seizure severity.

### 3.2 Chi Attenuates Epileptogenesis in the KA‐Induced Mouse Model

**FIGURE 2:**
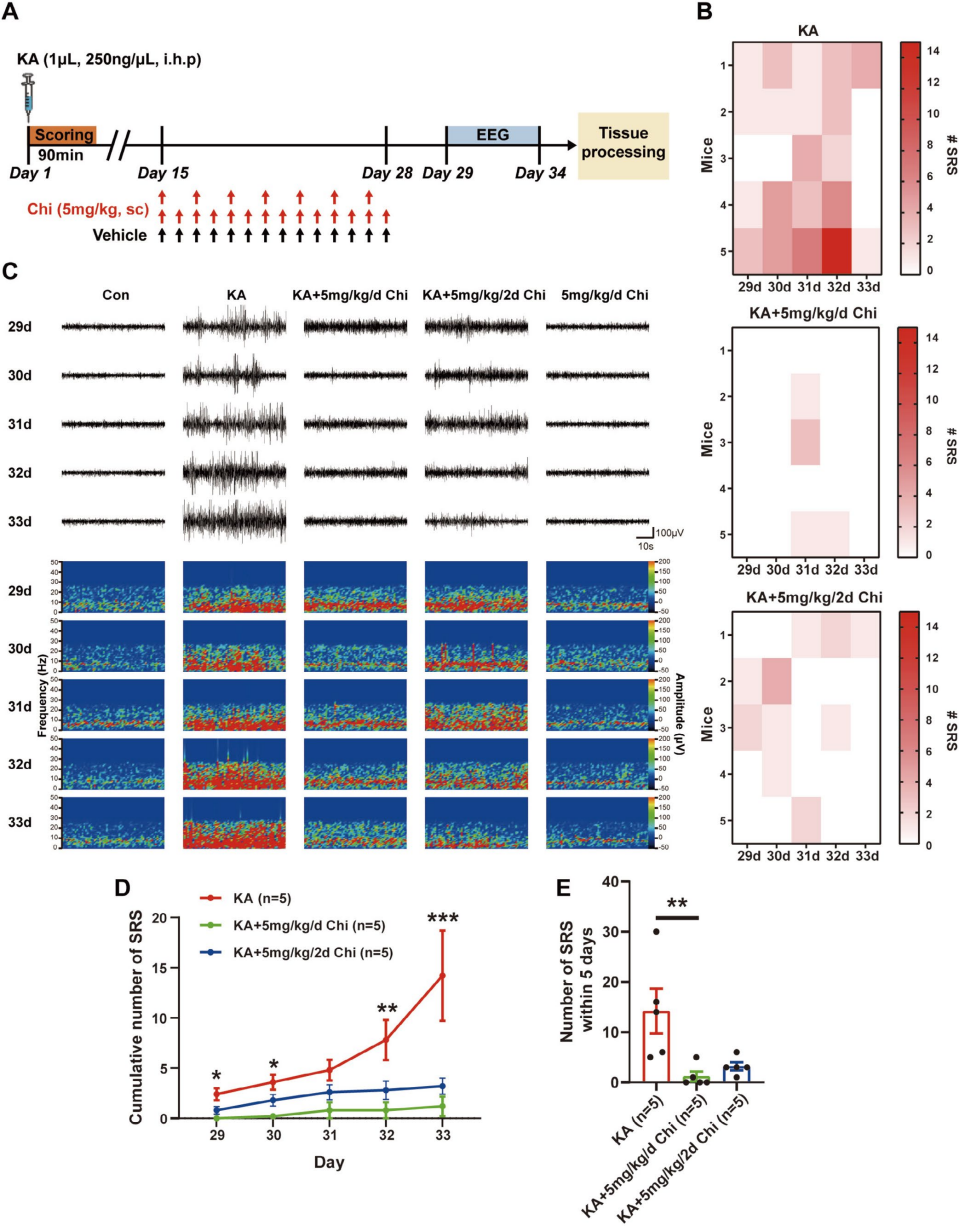
Chi attenuates epileptogenesis in the KA‐induced mouse model. (A) Schematic of the experimental protocol, including KA injection, chidamide treatment, EEG monitoring, and tissue collection. (B) Representative EEG traces showing seizure activity over the 5‐day recording period. (C) Heatmap displaying daily SRS frequency for each group of mice. (D) Quantification of average daily SRS events. (E) Cumulative SRS counts, comparing different chidamide dosing regimens. *p < 0.05, **p < 0.01, ***p < 0.001.

Regarding the critical role of epileptogenesis in epilepsy, anti‐epileptogenic therapy can serve as a novel strategy for treating patients. As such, we further assessed whether Chi blocked epileptogenesis by analyzing the frequency of SRS, a reliable indicator of epileptogenesis [23]. The overall experimental design is outlined in Figure 2A. The results of EEG recording demonstrated that KA‐treated mice exhibited frequent and robust SRS, while Chi treatment markedly reduced both the frequency and intensity of SRS events (Figure 2B). Heatmap analysis of individual seizure events across 5 days also revealed a marked reduction in the number of SRS in Chi‐treated groups compared to the KA group (Figure 2C). Quantitative analysis demonstrated a significant decrease in cumulative SRS number in both Chi‐treated groups, with daily administration showing greater efficacy (Figure 2D). Similarly, the total number of SRS events within the 5‐day recording period was significantly lower in the Chi‐treated groups than that in the KA group (Figure 2E). Collectively, these results indicate that Chi effectively suppresses the progression of epileptogenesis by reducing SRS frequency and severity, with daily administration providing superior seizure control.

### 3.3 Chi Preserves Neuronal Survival, Reduces Mossy Fiber Sprouting and Glial Activation in a KA‐Induced Epileptogenesis Model

**FIGURE 3:**
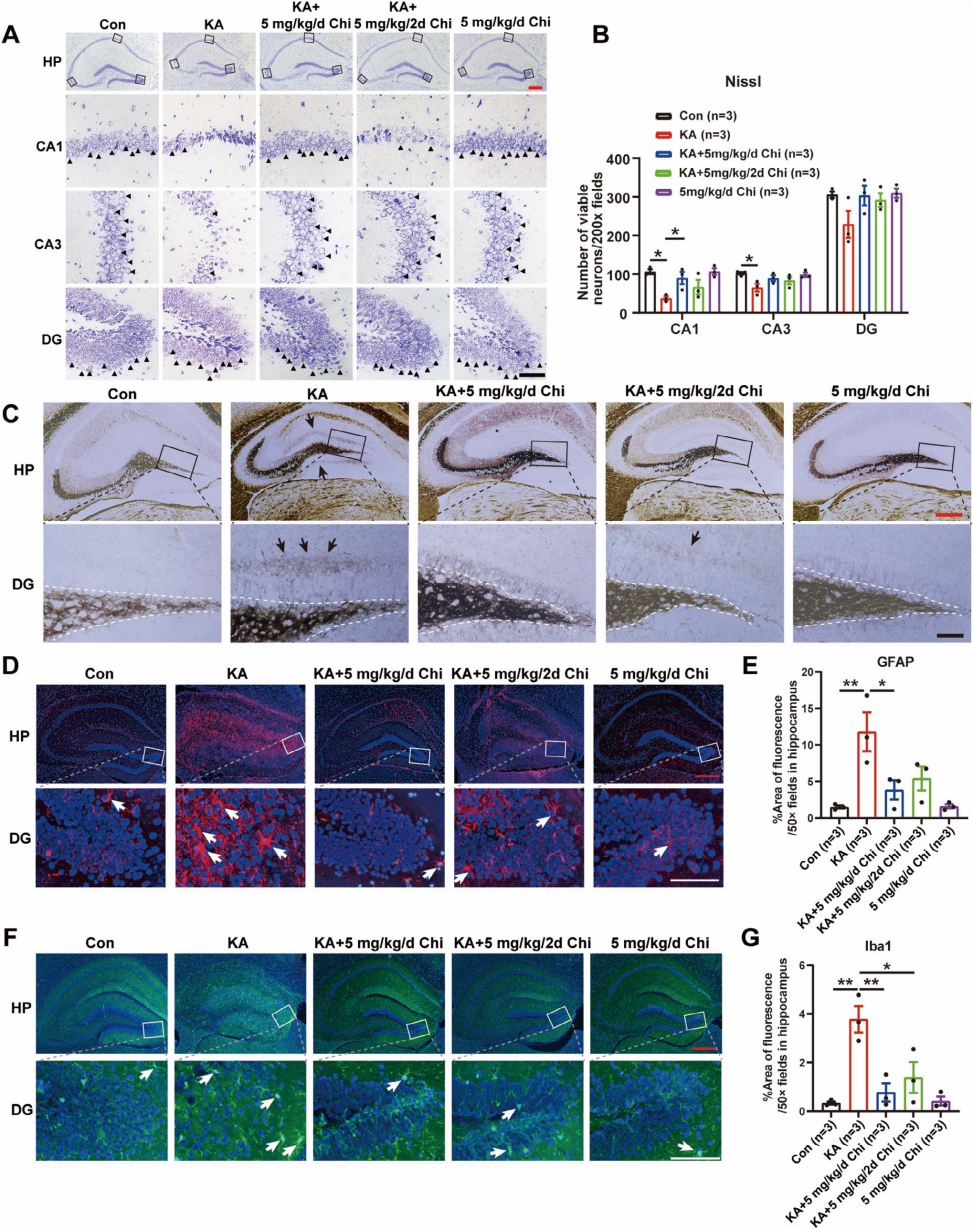
Chi preserves neuronal survival, reduces mossy fiber sprouting and glial activation in the KA‐induced epileptogenesis model. (A) Representative images of Nissl staining in the hippocampus. Black triangles indicate normal neurons. (B) Quantification of surviving neurons based on Nissl staining. (C) Representative images of mossy fiber sprouting visualized by Timm staining. The black arrow indicates the mossy fiber sprouting. (D) Representative images of GFAP immunostaining for astrocyte activation. (E) Quantification of GFAP‐positive area. (F) Representative images of Iba1 immunostaining for microglial activation. (G) Quantification of the Iba1‐positive area. Red scale bar: 100 μm, black scale bar: 25 μm. *p < 0.05, **p < 0.01, ***p < 0.001.

Since neuronal loss, especially in the hippocampus, is a common phenomenon during epileptogenesis [24], the effect of Chi on neuronal damage was further assessed in our present work, as summarized in Figure 2A. It was demonstrated that daily administration of Chi (5 mg/kg) for 14 consecutive days markedly alleviated pyramidal neuronal damage in the CA1 region during epileptogenesis. Additionally, a trend toward reduced neuronal damage was also observed in the CA3 and dentate gyrus (DG) regions (Figure 3A,B). With respect to MFS, a well‐known pathological marker following epileptogenesis, the results of Timm staining showed that daily administration of Chi significantly attenuated MFS in the DG region of the KA mouse model (Figure 3C), despite a less pronounced effect being found when Chi was administered every other day. Altogether, these results indicate that treatment with Chi effectively suppresses epileptogenesis‐associated events such as neuronal loss and MFS. Moreover, glial cell activation, namely, astrogliosis and microgliosis, is also a well‐established pathological hallmark of epileptogenesis, contributing to chronic neuroinflammation and facilitating recurrent seizures [25, 26]. Therefore, the effect of Chi on glial cell activation was also evaluated. The results of immunofluorescence staining for GFAP and Iba1, which are well‐known molecular markers of astrocytes and microglia, respectively, demonstrated that astrogliosis (Figure 3D,E) and microgliosis (Figure 3F,G) occurred in the hippocampus 1 month after KA injection. Notably, daily administration of Chi markedly attenuated this phenomenon. These findings suggest that Chi can effectively inhibit glial activation during epileptogenesis.

### 3.4 Chi Reduces Neuronal HDAC1 Expression in the KA‐Induced Epilepsy Model

**FIGURE 4:**
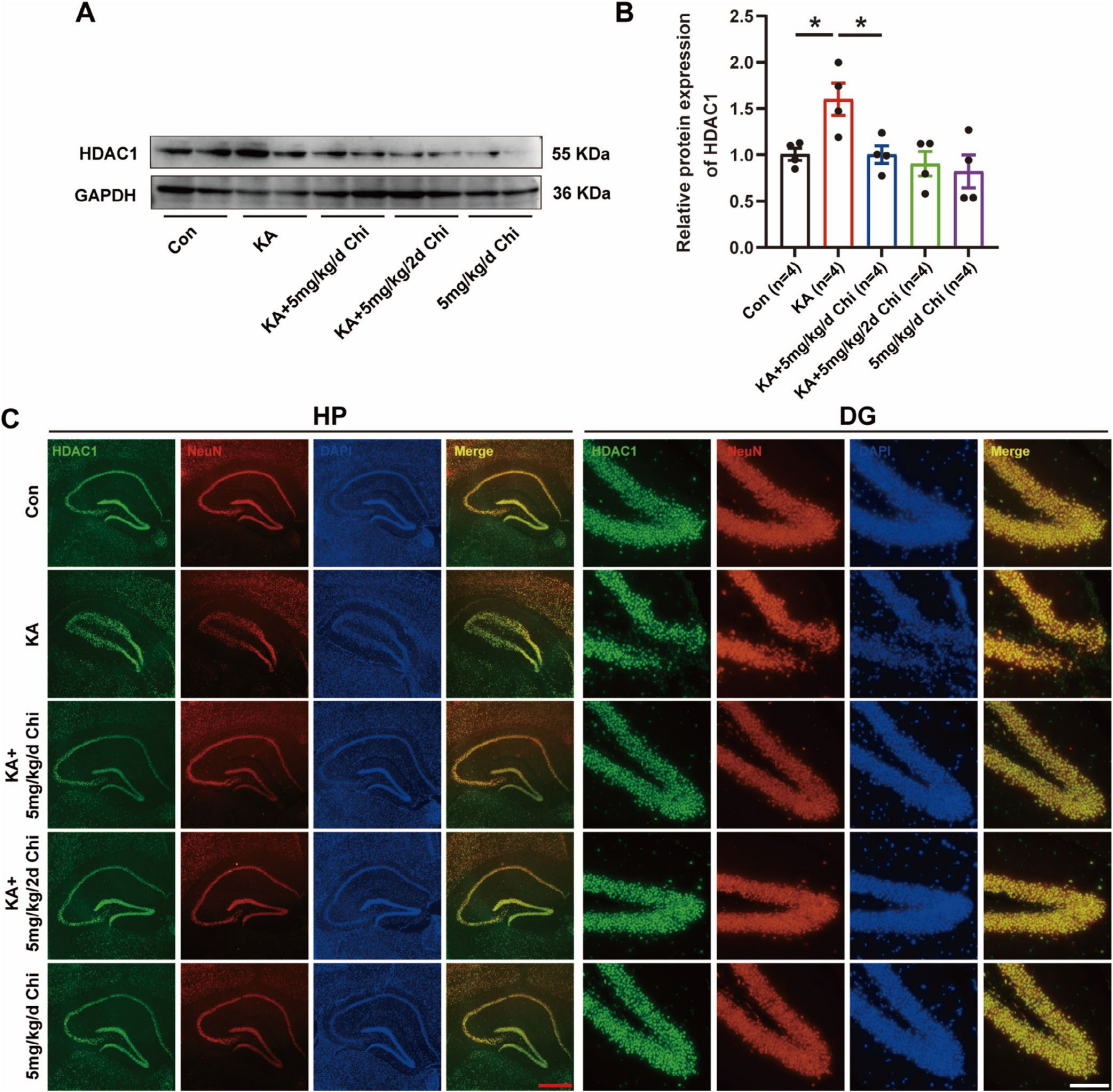
Chi reduces neuronal HDAC1 expression in the KA‐induced epilepsy model. (A) Representative Western blot (WB) image showing HDAC1 protein expression. (B) Quantification of HDAC1 protein levels based on WB analysis. (C) Representative immunofluorescence images showing co‐localization of HDAC1 with NeuN in the hippocampus and DG region. Red scale bar: 500 μm, black scale bar: 100 μm. *p < 0.05.

Previous evidence showed that HDAC1 is an important target of Chi [27]. To determine whether Chi modulates HDAC1 expression in the context of epilepsy, we first assessed HDAC1 protein levels in the hippocampus of KA‐induced epileptic mice. Western blot analysis revealed a significant upregulation of HDAC1 expression following KA injection, which was notably reduced after daily Chi treatment (Figure 4A,B). To further characterize the cell‐type specificity of HDAC1 expression, we performed double immunofluorescence staining for HDAC1 and the neuronal marker NeuN (Figure 4C). The results showed that daily administration of Chi reversed KA‐induced hippocampal deformation and preserved hippocampal integrity. Moreover, HDAC1 immunoreactivity was predominantly localized to NeuN‐positive neurons. These findings indicate that Chi specifically downregulates HDAC1 expression in hippocampal neurons and protects against hippocampal damage, which may contribute to its anti‐epileptogenic effects.

### 3.5 Neuronal HDAC1 Overexpression Abrogates the Anti‐Epileptogenic Effect of Chi

**FIGURE 5:**
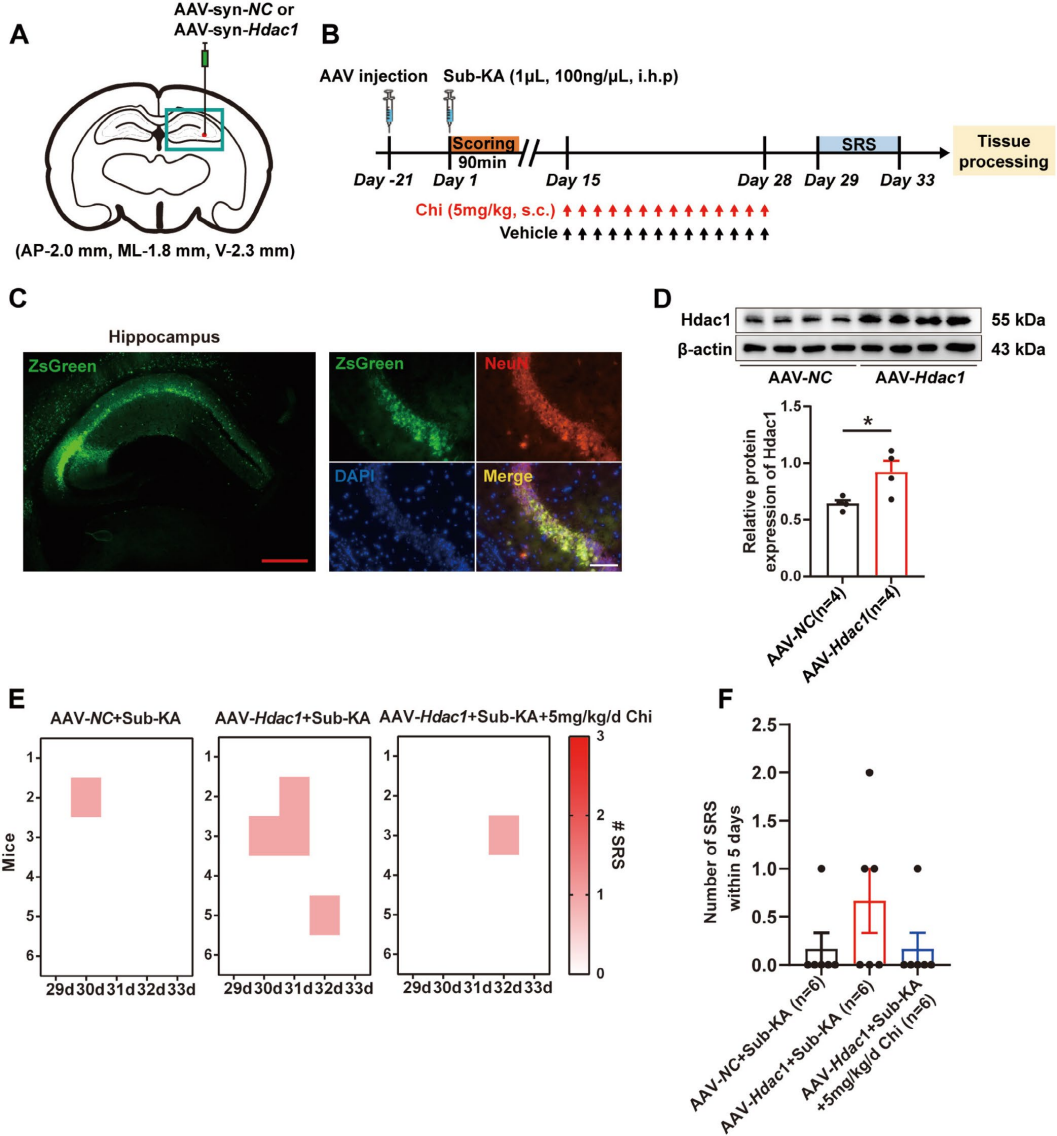
Neuronal *HDAC1* overexpression abrogates the anti‐epileptogenic effect of Chi. (A) Schematic of AAV‐mediated *HDAC1* overexpression in neurons. (B) Experimental timeline including viral injection, KA induction, and chidamide treatment. (C) Spontaneous fluorescence of ZsGreen in the hippocampus after AAV injection, and representative immunofluorescence images showing co‐localization of ZsGreen expression with NeuN. (D) Representative Western blot showing HDAC1 overexpression, and quantification of HDAC1 protein levels. (E) Heatmap of daily SRS events per mouse. (F) Cumulative SRS events over the 5‐day EEG monitoring period. Red scale bar: 500 μm, black scale bar: 100 μm. *p < 0.05.

To further investigate whether neuronal HDAC1 can affect the anti‐epileptogenic effect of Chi, HDAC1 overexpression was conducted in neurons via AAV delivery under the control of neuron‐specific promoter Syn, as summarized in Figure 5A. The experimental design and timeline are illustrated in Figure 5B. Spontaneous fluorescence of ZsGreen in the hippocampus confirmed successful viral transduction, as evidenced by co‐localization of ZsGreen with NeuN in the hippocampus (Figure 5C), indicating successful entry of the virus into neurons. Western blot analysis further verified a robust increase in HDAC1 protein levels in the hippocampus following AAV‐mediated gene transfer (Figure 5D). Furthermore, in vivo HDAC1 overexpression in neurons exacerbated SRS of mice succumbing to a sub‐convulsive dose of KA (Sub‐KA). However, treatment with Chi evidently reversed this phenomenon (Figure 5E,F). These findings suggest that neuronal HDAC1 overexpression exacerbates SRS occurrence during epileptogenesis and further support the notion that the antiepileptogenic effect of Chi is likely mediated, at least in part, through suppression of HDAC1 in neurons.

### 3.6 Neuronal HDAC1 Overexpression Abrogates Inhibitory Effects of Chi on Epileptogenesis‐Associated Pathogenic Events

**FIGURE 6:**
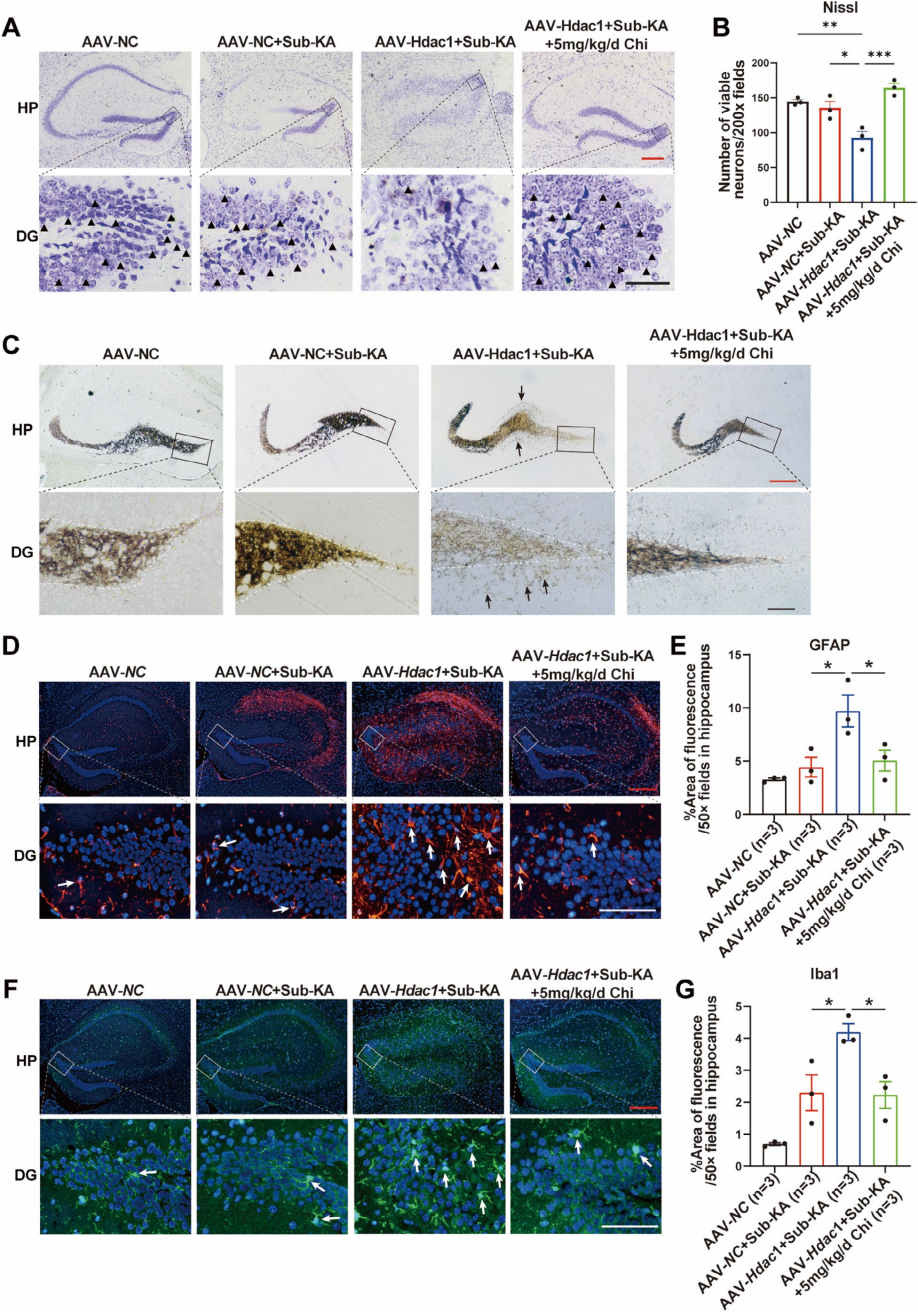
Neuronal *HDAC1* overexpression abrogates inhibitory effects of Chi on epileptogenesis‐associated pathogenic events. (A) Representative images of Nissl staining in the hippocampus. Black triangles indicate normal neurons. (B) Quantification of surviving neurons based on Nissl staining. (C) Representative images of mossy fiber sprouting visualized by Timm staining. The black arrow indicates the mossy fiber sprouting. (D) Representative images of GFAP immunostaining for astrocyte activation. (E) Quantification of GFAP‐positive area. (F) Representative images of Iba1 immunostaining for microglial activation. (G) Quantification of the Iba1‐positive area. Red scale bar: 100 μm, black scale bar: 25 μm. *p < 0.05, **p < 0.01, ***p < 0.001.

To evaluate the effect of neuronal HDAC1 overexpression on Chi‐mediated neuroprotection, we first examined neuronal survival in the hippocampus using Nissl staining. Sub‐KA administration did not cause significant neuronal loss compared with controls, whereas overexpression of neuronal HDAC1 exacerbated neuronal loss. Chi treatment significantly increased the number of viable neurons (Figure 6A,B). Next, no significant MFS was observed in the Sub‐KA group, while robust MFS was found after overexpression of HDAC1 in neurons, which was significantly inhibited by Chi treatment (Figure 6C). Furthermore, we also assessed whether neuronal HDAC1 overexpression could affect the inhibitory effects of Chi on glial cell activation following epileptogenesis. It was obvious that astrocyte activation in the hippocampus was found in Sub‐KA‐treated mice with neuronal HDAC1 overexpression, which was markedly inhibited after Chi treatment, as reflected by a decreased GFAP‐positive area (Figure 6D,E). Similarly, overexpression of HDAC1 in neurons after Sub‐KA administration induced pronounced microglial activation as indicated by Iba1 immunostaining. Treatment with Chi dramatically reversed this phenomenon (Figure 6F,G).

## 4 Discussion

In this study, we provide the first evidence that Chi, an FDA‐approved histone deacetylase inhibitor widely used in oncology, exerts an anti‐epileptogenic effect in epilepsy mouse models. Although Chi has demonstrated therapeutic benefits in various malignancies [28, 29, 30] and, more recently, preclinical neurodegenerative models such as Alzheimer's disease [31], its role in epileptogenesis has not previously been explored. By combining a systematic screen of clinically available histone deacetylase inhibitors with in vivo validation, Chi was screened to markedly improve neuronal survival and attenuate both seizure susceptibility and epileptogenesis in chemically induced mouse models. Our data further suggest that these effects are at least partly mediated through selective inhibition of HDAC1 in neurons, as overexpression of neurons evidently reversed the anti‐epileptogenic effect of Chi. These findings extend the therapeutic potential of Chi beyond oncology and highlight its promise as a repurposed, disease‐modifying agent for epilepsy.

Seizure susceptibility is a critical determinant of disease burden and progression in epilepsy, reflecting the underlying hyperexcitability and network remodeling that drive recurrent attacks [9]. Elevated susceptibility not only predicts the severity and frequency of seizures but also correlates with long‐term cognitive deficits and other comorbidities [32]. Therefore, reducing seizure susceptibility is considered an important step toward genuine disease modification. In our present study, Chi markedly suppressed seizure susceptibility in both PTZ‐ and KA‐induced seizure models, indicating that its anti‐seizure effects can further modulate the epileptogenic process.

Epileptogenesis is commonly associated with the development of genetic or acquired epilepsy and may continue for a prolonged period after the initial diagnosis. It represents a dynamic process involving complex molecular and structural alterations that drive the transition of the brain from a normal state to chronic hyperexcitability [33]. Key mechanisms include neurodegeneration, aberrant neurogenesis, gliosis, abnormal synaptic remodeling such as mossy fiber sprouting, blood–brain barrier disruption, recruitment of inflammatory cells, and ion channel dysfunction [9, 10]. Together, these changes promote recurrent spontaneous seizures and disease progression. These advances underscore epileptogenesis as a dynamic and therapeutically tractable process. Within this broader therapeutic landscape, pharmacological modulation remains a particularly attractive strategy due to its translational feasibility and potential for early intervention. Our findings show that Chi markedly suppresses epileptogenesis in a KA‐induced chronic epilepsy model, as reflected by reduced seizure development and progression. These results suggest that beyond its acute anti‐seizure effects, Chi can also modify the underlying disease process and thus holds promise as a novel anti‐epileptogenic therapy.

Neuronal loss, glial activation, and aberrant mossy fiber sprouting are widely recognized as key pathological features of the epileptogenic process and play critical roles in the initiation and progression of epilepsy. Neuronal loss, particularly in the hippocampus, disrupts inhibitory circuits and facilitates hyperexcitability, thereby lowering the seizure threshold [34]. Concurrently, activated astrocytes and microglia contribute to a pro‐inflammatory microenvironment through the release of cytokines, chemokines, and reactive oxygen species, which further exacerbate neuronal dysfunction and network instability [35, 36]. In addition, aberrant mossy fiber sprouting leads to the formation of recurrent excitatory synaptic circuits in the dentate gyrus, reinforcing epileptic network synchronization and seizure propagation [37]. These structural and cellular changes disrupt normal network homeostasis, promote excitatory‐inhibitory imbalance, and are strongly linked with poor treatment outcomes [9]. Interventions capable of mitigating these pathological processes are therefore considered to be vital for achieving a disease‐modifying effect. In our study, Chi treatment markedly attenuated neuronal loss, reduced astroglial and microglial activation, and suppressed mossy fiber sprouting in a KA‐induced epilepsy mouse model.

As a well‐characterized HDAC inhibitor, Chi has been shown to selectively target class I HDACs, thereby influencing a broad range of biological processes, including cell cycle regulation, inflammation, and synaptic plasticity. Increasing evidence implicates HDACs in the pathogenesis of epilepsy, where dysregulated HDAC activity can alter gene transcription, promote neuroinflammation, and contribute to maladaptive network remodeling [21]. Altered expression and activity of class I HDACs, including HDAC1, have been reported in both human epileptic hippocampal tissues and experimental models of temporal lobe epilepsy, indicating that HDAC‐dependent transcriptional dysregulation contributes to pathological network remodeling associated with epilepsy development [20, 38, 39]. In resected hippocampal samples from patients with mesial temporal lobe epilepsy accompanied by hippocampal sclerosis, HDAC1 expression and nuclear localization are significantly increased, suggesting its involvement in aberrant gene regulation during chronic epilepsy [38]. Moreover, in KA‐ and Pilo‐induced epilepsy models, class I HDAC expression undergoes dynamic changes across different stages of epileptogenesis, further supporting a role for these enzymes in the progression of epileptogenesis [39]. Functionally, pharmacological inhibition of HDAC activity targeting class I HDACs has been shown to delay epilepsy development, reduce seizure persistence, and attenuate neuronal damage in experimental models, highlighting HDAC‐mediated deacetylation as a critical driver of epileptogenic processes [20]. In the present study, Chi was screened to potently improve neuronal survival in the Mg^2+^‐free‐induced PC12 cell model using an FDA‐approved HDAC inhibitor library and selectively suppress HDAC1 expression in the KA‐induced epilepsy model, coinciding with its anti‐seizure and anti‐epileptogenic effects. These results suggest that HDAC1 inhibition may represent a key mechanism by which Chi exerts neuroprotective and disease‐modifying actions in epilepsy and highlight the potential of isoform‐specific HDAC targeting to achieve greater efficacy with fewer off‐target effects.

Several limitations of the present study should be acknowledged. Clinically, Chi is generally well tolerated in cancer patients, with most adverse events being mild to moderate and reversible, and no consistent neurotoxicity has been reported [40]. Preclinical studies have further demonstrated that Chi can penetrate the blood–brain barrier, supporting its potential central nervous system activity [41]. Nevertheless, long‐term administration in epilepsy may involve distinct, potentially lower dosing regimens and sustained epigenetic modulation; therefore, further dose optimization and long‐term safety evaluations are necessary prior to clinical translation. Interestingly, Chi exhibited a stage‐dependent efficacy pattern, with intermittent administration appearing more effective during the early phase, whereas daily dosing showed greater benefits during the later chronic stage. This phenomenon likely reflects the dynamic and temporally evolving nature of epileptogenesis, during which molecular and epigenetic responsiveness varies over time [9, 42]. Early epileptogenic processes may thus be more sensitive to transient epigenetic modulation, while sustained pathological remodeling in the chronic phase may require continuous pharmacological engagement. Although our findings demonstrate that Chi attenuates neuronal injury and neuropathological remodeling in epilepsy models, the precise molecular pathways underlying these effects remain to be elucidated. In particular, it is unclear whether HDAC1 inhibition directly regulates neuronal loss, glial activation, and mossy fiber sprouting, or whether these changes occur secondary to broader network modulation. Moreover, the long‐term efficacy, durability, and safety of Chi in chronic epilepsy models warrant systematic investigation. Addressing these issues will be essential to clarify the mechanistic link between HDAC1 inhibition and neuropathological alterations and to determine the translational potential of Chi as a disease‐modifying therapy for epilepsy.

## 5 Conclusions

In summary, our findings illustrate that Chi, an important HDAC inhibitor, improves diverse facets involving epileptogenesis, which include reduction of seizure susceptibility, attenuation of neuronal survival, and inhibition of glial activation and mossy fiber sprouting. It exerts an anti‐epileptogenic effect, possibly via inhibiting neuronal HDAC1, as overexpression of HDAC1 under the control of neuron‐specific promoter Syn evidently blocks Chi's effect. These evidences indicate that Chi serves as a promising anti‐epileptogenic drug in the future.

## Author Contributions

Xiao‐Yuan Mao initiated the project. Qian Guo and Zhao‐Jun Wang performed the experiments. Qian Guo, Zhao‐Jun Wang, Wei‐Bo Dong, and Li Pang conducted the data analysis. Qian Guo drafted the manuscript. Xiao‐Yuan Mao revised the manuscript. All authors read and approved the final version of the manuscript.

## Funding

This work was supported by the National Natural Science Foundation of China, 82274027, 82474014, and Natural Science Foundation of Hunan Province, 2025JJ20087.

## Disclosure

All experimental procedures were approved by the Institutional Animal Care and Use Committee of Central South University (approval number: CSU‐2024‐0212) and were conducted in strict accordance with the National Institutes of Health Guide for the Care and Use of Laboratory Animals. The authors declare that they have no competing interests.

## Conflicts of Interest

The authors declare no conflicts of interest.

## Data Availability

The data that support the findings of this study are available from the corresponding author upon reasonable request.
